# Prone retroperitoneal robotic-assisted laparoscopic pyeloplasty for ureteropelvic junction obstruction

**DOI:** 10.3389/fsurg.2026.1730936

**Published:** 2026-03-11

**Authors:** Xiao Yang, Haonan Chen, Hao Yu, Zhengye Tan, Lingkai Cai, Qiang Cao, Qiang Lu

**Affiliations:** Department of Urology, The First Affiliated Hospital of Nanjing Medical University, Nanjing, China

**Keywords:** prone position, pyeloplasty, retroperitoneal, robotic, ureteropelvic junction obstruction

## Abstract

**Objective:**

To evaluate the feasibility and preliminary outcomes of prone retroperitoneal robotic-assisted laparoscopic pyeloplasty (prRALP) for ureteropelvic junction obstruction (UPJO), an innovative approach designed to optimize surgical exposure and suturing.

**Methods:**

This retrospective cohort study analyzed four patients who underwent prRALP between September 2023 and May 2024. The surgical technique involved prone positioning and multi-port robotic access, enabling direct posterior exposure of the renal pelvis and proximal ureter. Primary outcomes included operation time (OT), estimated blood loss (EBL), complications (Clavien-Dindo classification), and postoperative renal function (eGFR). Success was defined by radiographic resolution of obstruction, symptom relief, and no need for reintervention.

**Results:**

All procedures were completed robotically without open conversion and reoperation. Mean OT was 64.6 ± 14.4 min, with minimal blood loss of 27.5 ± 15.0 mL and no transfusions. The mean postoperative hospital stay was 3.3 ± 0.5 days. One minor complication (fever, Clavien 1) occurred (25%). Postoperative eGFR improved by 1.3 ± 14.2 mL/min/1.73 m^2^ at 90-day follow-up, with all patients achieving obstruction-free recovery and a mean eGFR of 87.2 ± 69.8 mL/min/1.73 m^2^ being maintained at 1-year postoperatively.

**Conclusion:**

prRALP demonstrates feasibility and safety, leveraging prone position to enhance retroperitoneal access and suturing precision. Larger prospective studies are warranted to validate its technical benefits and reproducibility.

## Introduction

Robotic-assisted laparoscopic pyeloplasty (RALP) has become the mainstream surgical intervention for ureteropelvic junction obstruction (UPJO) since its initial description by Gettman et al. in 2002 (1, 2). Compared to conventional laparoscopic approaches, RALP offers superior instrument maneuverability and enhanced visualization, thereby improving surgical precision and shortening learning curve (2). Currently, two principal surgical approaches exist: transperitoneal and retroperitoneal. While both methods demonstrate comparable success rates, operation time, and complication profiles (3), the retroperitoneal approach offers distinct advantages, including faster return to diet, shorter drain duration, and reduced hospital stay (3, 4). These benefits are attributed to its ability to avoid bowel manipulation and minimize peritoneal irritation, thereby facilitating more rapid postoperative recovery.

Recently, several reports successfully introduced retroperitoneal single-port (SP) RALP via supine low anterior access (LAA) (5, 6). Compared to traditional transperitoneal approach, SP-LAA may help optimize operating room time, promote faster patient recovery, and reduce postoperative opioid use (6). Nevertheless, SP-LAA pyeloplasty demands advanced surgical skills for confined-space instrument maneuvering, and its adoption remains limited by the scarcity of SP systems in many centers. Therefore, the reproducibility of this technique requires further validation. In our preliminary exploration of prone retroperitoneal robotic-assisted laparoscopic radical nephroureterectomy (prRARNU) (7) and partial nephrectomy (prRAPN) (8), we observed distinct advantages in achieving direct access to the renal hilum. Given that the renal pelvis lies posterior to the hilar vessels anatomically, we hypothesized that prone position could further optimize exposure for pyeloplasty. Building upon these findings, we introduce an innovative prone retroperitoneal RALP (prRALP) technique, detailing our surgical methodology and providing preliminary outcome analysis.

## Patients and methods

### Study population and measurements

This retrospective cohort study reviewed consecutive patients who underwent prRALP at in our center from September 2023 to May 2024. Demographic, perioperative, and follow-up data were collected. Primary outcomes included operation time (OT), estimated blood loss (EBL), transfusion and reoperation rate, conversion rates to open surgery, complication rates (Clavien-Dindo classification), postoperative estimated glomerular filtration rate (eGFR) and length of stay.

### Surgical techniques

**Figure 1:**
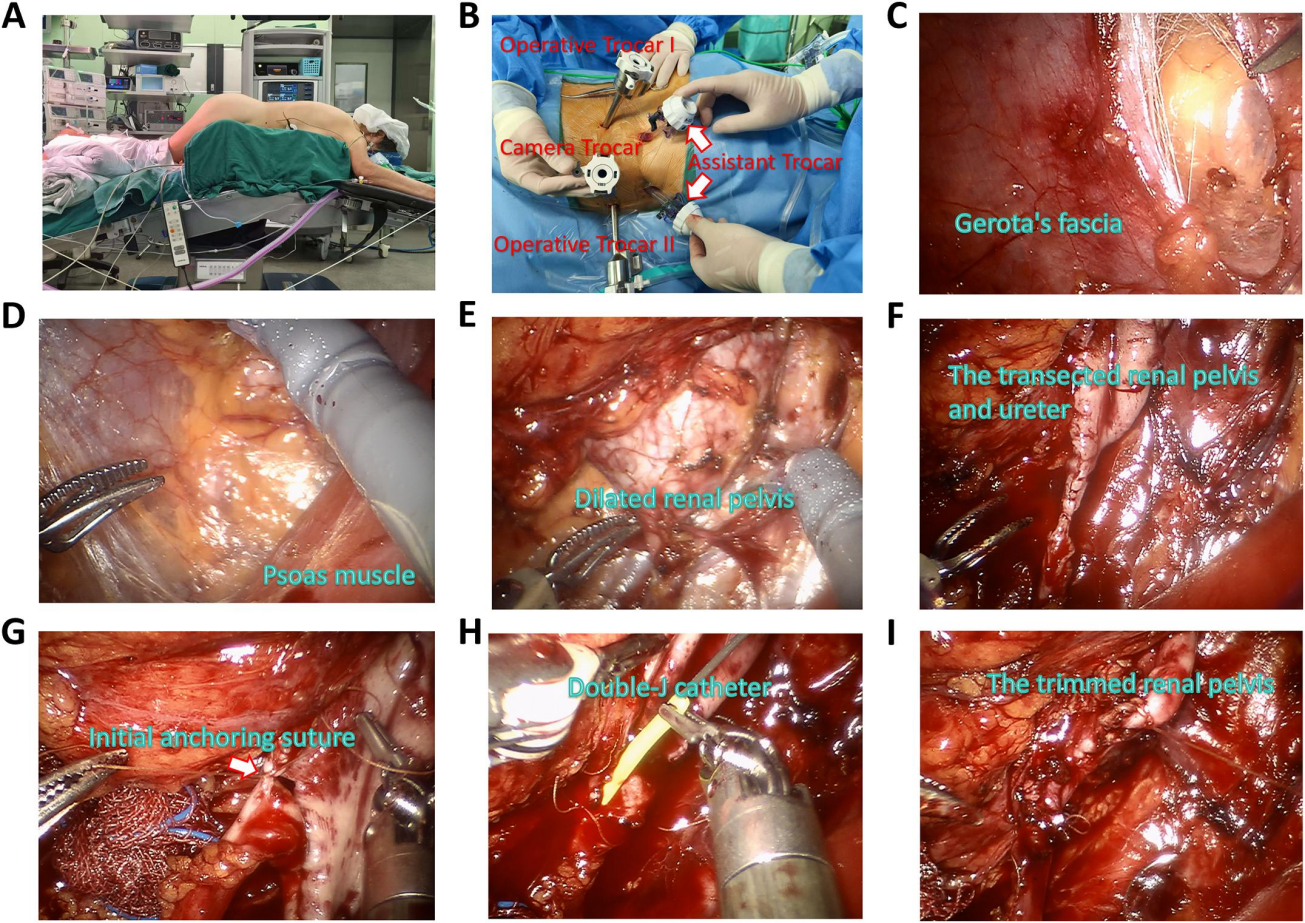
Patient positioning, trocar placement, and surgical technique for prRALP. **(A)** The prone ‘jackknife’ position for patient. **(B)** Port arrangement. **(C)** Longitudinally opening the Gerota's fascia. **(D)** Dissection of retroperitoneal fat along the psoas muscle. **(E)** Exposure of the dilated renal pelvis. **(F)** Transection of the renal pelvis and ureter at both the proximal and distal narrow segments, while maintaining continuity. **(G)** Placement of the initial anchoring suture between the renal pelvis and ureteral stump to ensure proper alignment without torsion. **(H)** Placement of a Double-J catheter. **(I)** Trim and suture the renal pelvis.

The patient's position and trocar placement were similar to those used in prRAPN (Figures 1A,B) (8). The operative field was divided into three equal zones along the posterior midline-to-midaxillar*y* axis, with the medial boundary corresponding to the paraspinal muscles and the lateral boundary aligned with the costal apex of the 12th rib. A 3-cm skin incision was created along the lateral boundary between the L2–L3 interspace. After blunt separation of the muscle layers and transversalis fascia using Kelly clamps, the retroperitoneal space was developed by finger dissection. A self-made balloon dissector was then inflated to approximately 800 mL to expand the working space and facilitate ventral displacement of extraperitoneal fat.

An 8–12 mm camera port was introduced through the primary incision. One 8-mm robotic working port was placed 1–2 cm inferior to the camera port along the medial boundary, and a second 8-mm working port was positioned at the same horizontal level along the midaxillary line. One or two 12 mm assistant ports were routinely placed superior to the iliac crest to optimize instrument access. The surgical procedure is as follows:

Dissection and exposure of the renal pelvis and upper ureter: The Gerota's fascia is opened (Figure 1C), and the retroperitoneal fat is dissected along the psoas muscle to expose the dilated renal pelvis and the renal pelvis-ureteral junction (Figures 1D,E).
Resection of the renal pelvis and ureter: The renal pelvis and ureter are transected at both the proximal and distal narrow segments, while maintaining continuity between the renal pelvis and ureteral segment (Figure 1F). The ureter is then longitudinally incised on the posterior-lateral side.
Suturing the posterior wall of the renal pelvis-ureter Junction: The initial anchoring suture is placed between the renal pelvis and ureteral stump to ensure proper alignment without torsion (Figure 1G). Subsequently, a complete transection of the renal pelvis and ureter is performed, followed by continuous anastomosis using absorbable fine sutures (4-0 or 5-0 Vicryl or Monocryl).
Placement of a Double-J catheter and anastomosis of the anterior wall: A Double-J catheter is inserted through the anastomosis (Figure 1H). The anterior wall is anastomosed using the same technique as the posterior wall, with concomitant appropriate trimming of the renal pelvis (Figure 1I).

### Follow-up

Complications were evaluated based on the Clavien-Dindo classification system. The Double-J catheter is removed 4–8 weeks postoperatively, with dynamic monitoring of eGFR. The postoperative surveillance included ultrasonography, computed tomography urography, or magnetic resonance urography, with retrograde urography performed when clinically indicated. Surgical success was defined by imaging evidence of unobstructed urinary drainage, exemplified in Supplementary Figure S1, together with symptom relief and no requirement for additional intervention.

## Results

Overall, the study cohort comprised 4 UPJO patients (75% male) with a mean age of 52.0 ± 21.3 years, and mean BMI of 24.9 ± 7.9 kg/m^2^. All patients were American Society of Anesthesiology class 2, with age-adjusted Charlson comorbidity index of 3.3 ± 2.2. Obstruction locations included pelvi-ureteric junction (75%) and proximal ureter (25%), equally distributed between left and right sides (50% each). All procedures were successfully completed without conversion to open surgery. The mean OT was 64.6 ± 14.4 min with EBL of 27.5 ± 15.0 mL. Perioperative laboratory changes showed a mean hemoglobin decrease of 9.8 ± 8.3 g/L, with no patients requiring transfusion. Patients experienced rapid recovery with a mean postoperative hospital stay of 3.3 ± 0.5 days. Baseline demographics and clinical characteristics of patients were shown in Table 1.

**Table 1:** Baseline demographics and clinical characteristics.

Parameter	Result
Age (year)^a^	52.0 ± 21.3
Gender, *n* (%)	
Male	3 (75.0)
Female	1 (25.0)
BMI (kg/m^2^)^a^	24.9 ± 7.9
ASA score = 2, *n* (%)	4 (100.0)
aCCI^a^	3.3 ± 2.2
Preoperative Hemoglobin (g/L)^a^	128.5 ± 12.4
Preoperative eGFR (mL/min/1.73 m^2^)^a^	83.9 ± 74.6
Obstruction location, *n* (%)	
Pelvi-ureteric junction	3 (75.0)
Proximal ureter	1 (25.0)
Laterality, *n* (%)	
Left	2 (50.0)
Right	2 (50.0)

ASA, American Society of Anesthesiologists; BMI, body mass index; aCCI, age-adjusted Charlson comorbidity index; CKD, chronic kidney disease.

^a^ Result reported as the mean ± standard deviation.

Renal function outcomes demonstrated a mean postoperative eGFR of 85.2 ± 72.2 ml/min/1.73 m^2^ at 90-day follow-up, representing an increase of 1.3 ± 14.2 ml/min/1.73 m^2^ compared to preoperative baseline values. Long-term renal function assessment at 1-year postoperatively demonstrated stable renal preservation, with a mean eGFR of 87.2 ± 69.8 mL/min/1.73 m^2^ being maintained. The complication rate was 25% (*n* = 1), consisting exclusively of minor events (fever, Clavien 1) without major complications or reoperations. At 3-month evaluation postoperatively, all patients showed radiographic evidence of improved urinary drainage with complete resolution of preoperative symptoms. Perioperative and follow-up outcomes were presented in Table 2.

**Table 2:** Perioperative and follow-up outcomes.

Parameter	Result
Operation time (min)^a^	64.6 ± 14.4
Estimated blood loss (mL)^a^	27.5 ± 15.0
*Δ*Hemoglobin (g/L)^a^	−9.8 ± 8.3
*Δ*eGFR (mL/min/1.73 m^2^)^a^	1.3 ± 14.2
eGFR in POD 90 (mL/min/1.73 m^2^)^a^	85.2 ± 72.2
eGFR in postoperative 1-year (mL/min/1.73 m^2^)^a^	87.2 ± 69.8
Transfusion, *n* (%)	0
Open surgery conversion, *n* (%)	0
Reoperation rate, *n* (%)	0
Cumulative postoperative complications in POD 90	
None	3 (75.0)
Minor (Clavien 1–2)	1 (25.0)
Postoperative length of stay (day)^a^	3.3 ± 0.5

*Δ* Hct, change in hematocrit between baseline and discharge; *Δ* eGFR, change in estimated glomerular filtration rate between baseline and POD 90; POD, postoperative day; NRS, numerical rating scale.

^a^ Result reported as the mean ± standard deviation.

## Discussion

RALP has solidified its role as the preferred minimally invasive treatment for UPJO since its inception, ongoing refinements focus on optimizing access, ergonomics, and recovery. While the retroperitoneal approach offers compelling advantages in avoiding peritoneal entry and bowel manipulation, thereby potentially accelerating recovery (9, 10), its execution in the conventional lateral decubitus position presents significant challenges. These include longer anatomic distance to the lesion (7), respiratory complications and potential nerve injuries (11), potentially increase the complexity of the procedure and extend operation time.

While recent innovations like supine anterior retroperitoneal access via a single-port platform represent alternative attempts to improve retroperitoneal ergonomics, their reproducibility and widespread applicability remain unvalidated. Our initial experience demonstrates that prRALP is a feasible and safe technique, which leverages the established benefits of multi-port robotic systems: superior 3D visualization, tremor filtration, and endo-wristed dexterity within an optimized retroperitoneal workspace.

By positioning the patient prone, gravity naturally retracts the abdominal organs and kidney ventrally, bringing the posteriorly situated ureteropelvic junction (UPJ) and proximal ureter directly into the surgeon's vision of manipulation. This approach achieves dual advantages: expanding the retroperitoneal working space while establishing direct posterior access to the target lesions, which is particularly advantageous for the pyeloplasty technique requiring meticulous posterior suturing. This anatomical advantage stands in stark contrast to the lateral approach, where achieving optimal exposure of the posterior UPJ often necessitates significant renal mobilization or awkward instrument positioning. Furthermore, the ventral displacement of retroperitoneal fat along Gerota's fascia directly exposes the fascia, thereby reducing the necessity for retroperitoneal fat dissection compared to the lateral position.

The anatomical advantages of the prone retroperitoneal approach make it distinctly suitable for specific conditions. Its primary indication remains UPJO, where the direct posterior access facilitates optimal exposure of the obstructed segment, and the renal pelvis for anastomosis. In our previous experience with prRARNU, we found that twist of the robotic joint was required to access the mid-to-distal ureteral segments (7). Interestingly, during prRALP procedures, we observed that the ureter above the L3 level could be managed directly without robotic arm twisting - a significant technical simplification. Therefore, prRALP is also well-suited for the repair of proximal ureteral strictures located above the level of the L3. Within this anatomical region, the ureter remains readily accessible within the retroperitoneal space in the prone position. The gravity-assisted exposure and robotic precision allow for effective identification of the stricture, meticulous dissection, and performance of a uretero-ureteral or ureteropelvic anastomosis. For strictures distal to L3, where pelvic anatomy limits visualization, alternative approaches (transperitoneal or LAA) may offer better ergonomics and exposure.

Although the prone position poses potential risks, including airway compression, accidental extubation, and intraoperative pressure injuries, urologists are generally familiar with renal anatomy in the prone position due to the routine use of percutaneous nephrolithotomy in this position. Recent study has also highlighted the application of the prone position in laparoscopic partial nephrectomy, showing no adverse effects on PaCO_2_, PaO_2_, or pH when compared to lateral partial nephrectomy (12). Furthermore, the safety of intraoperative ventilation in obese patients during prone positioning has been confirmed (13).

This report details our initial experience with prRALP. While promising, its retrospective nature, small cohort size, and lack of direct comparative data necessitate cautious interpretation. Future studies should focus on validating the effectiveness of prRALP in a larger, and prospective cohort, comparing it to the conventional lateral retroperitoneal approach, and assessing its impact on clinical outcomes, including operation time, complication rates, and long-term recovery. It is essential to determine whether the ergonomic and technical advantages of the prone position could translate into improved surgical outcomes and a lower incidence of postoperative complications.

## Conclusion

In conclusion, prRALP represents a promising advancement in the treatment of UPJO, potentially addressing some of the technical challenges associated with traditional retroperitoneal access. As the technique evolves and further evidence emerges, it may serve as a valuable addition to the surgical options for UPJO treatment, with the potential to improve patient outcomes and streamline the surgical process.

## Data Availability

The raw data supporting the conclusions of this article will be made available by the authors, without undue reservation.

## Supplementary material

Supplementary Figure S1 Preoperative and postoperative computed tomography images of a representative patient. **(A)** Preoperative CT demonstrating marked dilation of the left renal pelvis with hydronephrosis. **(B)** CT obtained at 2-year follow-up showing resolution of left hydronephrosis with no evidence of renal pelvic or ureteral obstruction.
