# Supplementary figures and images for: Prone retroperitoneal robotic-assisted laparoscopic pyeloplasty for ureteropelvic junction obstruction
Source: Front Surg. 2026 Mar 11;13:1730936. doi: 10.3389/fsurg.2026.1730936 (PMC13013488; doi:10.3389/fsurg.2026.1730936)

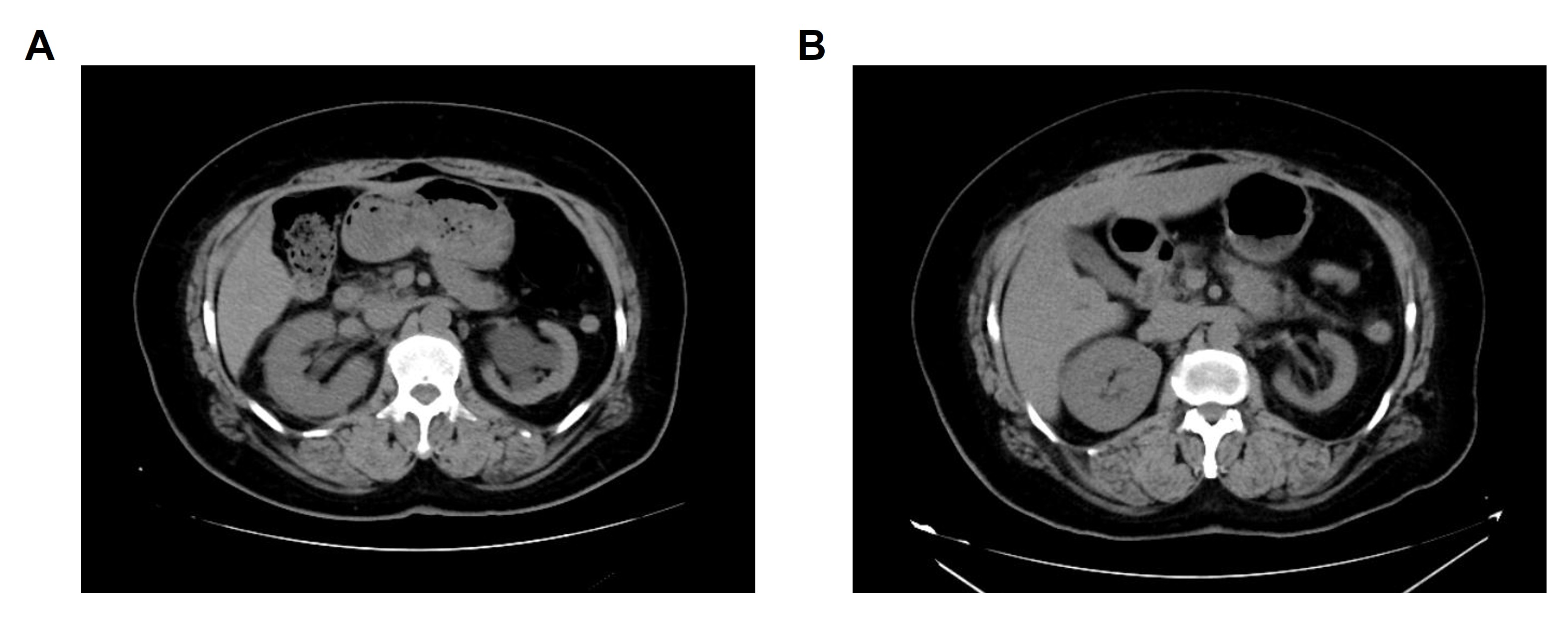

Supplement: Supplementary Figure S1 — Preoperative and postoperative computed tomography images of a representative patient. (A) Preoperative CT demonstrating marked dilation of the left renal pelvis with hydronephrosis. (B) CT obtained at 2-year follow-up showing resolution of left hydronephrosis with no evidence of renal pelvic or ureteral obstruction. [file Image1.jpeg]
